# Assessment of clinical efficacy of traditional Chinese medicine for the management of primary dysmenorrhea in the UK: A protocol of systematic review

**DOI:** 10.1097/MD.0000000000023246

**Published:** 2020-11-20

**Authors:** Ming-ming Fu, Xiang-dong Meng

**Affiliations:** a Department of English, Jiamusi College of Heilongjiang University of Chinese Medicine; b Department of Cardiology, Jiamusi University, Jiamusi, China.

**Keywords:** efficacy, primary dysmenorrhea, traditional Chinese medicine

## Abstract

**Background:**

This study aims to appraise the clinical efficacy of traditional Chinese medicine (TCM) for the management of patients with primary dysmenorrhea (PD) in the UK.

**Methods:**

We will comprehensively search electronic databases (Cochrane Library, PUBMED/MEDLINE, EMBASE, PsycINFO, AMED, Web of Science, and CNKI) and additional resources for original articles on randomized controlled trials published in English, Chinese, German, Spanish, Korean and Japanese. Outcomes will be the pain intensity, pain duration, menstrual cramps, amount of bleeding, and severity of dysmenorrhea symptoms, quality of life, and adverse events. Two authors will independently check all citations, extract data, and assess study quality. All potential conflicts will be solved through discussion by consulting another experienced author. A narrative synthesis will summarize the characteristics and findings of eligible trials. If it is possible, we will also pool the data and carry out meta-analysis.

**Results:**

The available evidence of the clinical efficacy of TCM for the treatment of PD in UK will be assessed through outcome measurements.

**Conclusion:**

The findings of this study will determine whether or not TCM is effective and safe for the treatment of PD in UK.

**OSF registration number:**

osf.io/jyc95.

## 1 Introduction

Primary dysmenorrhea (PD) is a prevalent gynecological health problem among adolescent girls and adult women.^[1–3]^ It is characterized by painful cramps in the lower abdomen during menstruation period.^[4,5]^ Patients with PD may also experience vomiting, nausea, diarrhea, fatigue, nervousness and dizziness.^[2–7]^ It is reported that its incidence varies from 45% to 72% of all reproductive age women; and this figure can reach as high as 93% of adolescent girls.^[8]^ If it can not be treated very well, it can prevent those people from participating in their daily activities.^[9,10]^ Although a variety of managements are reported to treat PD, their efficacy is still limited and some have severe adverse events.^[11–13]^ Thus, it is necessary to explore alternative therapy with fewer adverse events for treating PD.

Traditional Chinese medicine (TCM) has been used for the management of PD widely in China.^[14–17]^ It consists of herbal medicine, acupuncture, moxibustion, Tui-na, and any others.^[14–22]^ Studies suggested that TCM can manage PD in UK.^[23,24]^ However, there is still insufficient evidence to support the clinical efficacy and safety of TCM for PD in UK. Thus, this study will explore the clinical efficacy and safety of TCM for the treatment of PD in UK systematically and comprehensively.

## 2 Methods

### 2.1 Study registration

This study protocol was registered on OSF (osf.io/jyc95). It is being reported based on the guidelines of Preferred Reporting Items for Systematic Reviews and Meta-Analysis Protocol statement.

### 2.2 Eligibility criteria

#### 2.2.1 Types of population

We will include original randomized controlled trials (RCTs) of females in UK who had PD disorder, in spite of any race, gender, and economic status.

#### 2.2.2 Types of study

This study will only consider RCTs that investigated the clinical efficacy and safety of TCM in treating PD for inclusion. We will exclude any other studies, except RCTs.

#### 2.2.3 Types of exposure

We will include RCTs that utilized TCM (including Chinese herbal medicine, acupuncture, moxibustion, et al) as major treatment.

#### 2.2.4 Types of comparators

There is no restrictions to the control. However, we will exclude studies that used combined therapy with any forms of TCM.

#### 2.2.5 Types of outcomes

Outcomes are the pain intensity, pain duration, menstrual cramps, amount of bleeding, and severity of dysmenorrhea symptoms, quality of life, and adverse events.

### 2.3 Information sources

We will carry out a comprehensive search in electronic databases from the beginning to the current date (Cochrane Library, PUBMED/MEDLINE, EMBASE, PsycINFO, AMED, and Web of Science, and CNKI) and additional resources (reference lists of all included studies and relevant reviews, and websites of clinical trial registry) for original trials on RCTs published in English, Chinese, German, Spanish, Korean and Japanese. The search strategy for Cochrane Library is provided in Table 1. We will adapt similar search strategy for other electronic databases.

**Table 1:** Search strategy sample of Cochrane Library.

Number	Search terms
1	MeSH descriptor: (Dysmenorrhea) explode all trees
2	((dysmenorrhea^∗^) or (menstrual pain^∗^) or (painful menstruation^∗^) or (primary dysmenorrh^∗^) or (pain intensity^∗^)):ti, ab, kw
3	Or 1–2
4	(UK) explode all trees
5	((United Kingdom^∗^) or (Great Britain^∗^) or (Britain^∗^) or (Northern Ireland^∗^) or (U.K.^∗^)):ti, ab, kw
6	Or 4–5
7	(traditional Chinese medicine) explode all trees
8	MeSH descriptor: (acupuncture) explode all trees
9	MeSH descriptor: (electroacupuncture) explode all trees
10	MeSH descriptor: (acupuncture analgesia) explode all trees
11	MeSH descriptor: (acupuncture therapy) explode all trees
12	MeSH descriptor: (moxibustion) explode all trees
13	((Chinese medicine^∗^) or (herbal medicine^∗^) or (moxibustion^∗^) or (tui na^∗^) or (Qigong^∗^) or (cupping therapy^∗^) or (acupuncture^∗^) or (manual acupuncture^∗^) or (needling^∗^) or (acupionts^∗^) or (meridian^∗^) or (auricular needle^∗^) or (ear acupuncture^∗^) or (scalp acupuncture^∗^) or (abdominal acupuncture^∗^)):ti, ab, kw
14	Or 7–13
15	MeSH descriptor: (randomized controlled trials) explode all trees
16	((randomly^∗^) or (random^∗^) or (blind ^∗^) or (allocation ^∗^) or ((concealment^∗^) or (placebo ^∗^) or (sham^∗^) or (clinical^∗^) or (control^∗^) or (study^∗^) or (trial^∗^)):ti, ab, kw
17	Or 15–16
18	3 and 6 and 14 and 17

### 2.4 Selection process

Two authors will independently perform study selection process, respectively. First, titles and abstracts of all identified records will be screened, and all irrelevant studies will be removed. Then, full texts of potentially eligible articles will be read against all inclusion criteria and they will be determined to be included. Any divergence will be solved by discussion. We will present the selection process results in a flow chart.

### 2.5 Data extraction process

Two authors will independently extract data using a standardized form. For each included study, the following data will be extracted: title, authors, time of publication, location, study design, setting, and methods, study period, number of patients in each group, patient characteristics, interventions, controls, statistical methods, outcomes, main results/effect estimates, funding information, and conflict of interest. Any conflicts will be solved through discussion until consensus is reached. Missing or insufficient information will be obtained by contacting primary study authors via email or fax.

### 2.6 Study quality assessment

Two authors will independently rate study quality of included RCTs with respect to methodological criteria of Cochrane Risk of Bias Tool. It has 7 items, and each item is divided into 3 levels: low, unclear and high risk of bias. Any disagreements will be settled down by discussion with the help of a third author.

### 2.7 Quality of evidence assessment

Two authors will appraise quality of evidence for each outcome using Grades of Recommendation, Assessment, Development and Evaluation approach.^[25]^ Any divergence will be solved by a third author through discussion and a final decision will be made.

### 2.8 Statistical analysis

This study will conduct statistical analysis using RevMan 5.3 software. The results of quantitative trials will be estimated as frequencies, percentages, means, relative risks, or odds ratios with 95% confidence intervals. The results of qualitative trials will be presented according to the descriptive characteristics and main themes. We will check statistical heterogeneity across included RCTs using *I*^2^ test. Value of *I*^2^ *≤* 50% means homogeneity, and we will use a fixed-effects model to pool the data. Value of *I*^2^ *>* 50% exerts obvious heterogeneity, and we will utilize a random-effects model to synthesize the data. If sufficient data on the same outcome measurement are available, we will plan to carry out a meta-analysis based on the enough similarity in study information, patient characteristics, interventions, and controls. If it is not possible to perform a meta-analysis, we will narratively synthesize outcome data, and characteristics and results of included RCTs will be descriptively summarized in evidence tables.

In addition, we will undertake a subgroup analysis to investigate the possible factors that may result in obvious heterogeneity based on the different study information, patient characteristics, treatments, and comparators. If necessary, we will carry out a sensitivity analysis to examine the stability of merged results by eliminating low quality RCTs. If more than 10 studies on the same outcome are included, we will perform a funnel plot and Egger's regression test to identify if there is any possible reporting bias.^[26,27]^

### 2.9 Ethics and dissemination

This study protocol does not need ethical approval since it is conducted based on published data. It will be published on a peer-reviewed journal or a conference meeting.

## 3 Discussion

This study aims to assess the clinical efficacy of TCM in treating PD in UK population. So far, there is no up-to-date analysis that specifically merges results from different RCTs on TCM for PD systematically and comprehensively. Thus, it is very necessary to explore this issue so that insights can be utilized to inform clinical practice and decision makers. However, there may be several limitations exist in this study. First, this study only focuses on UK population, which may limit the number of eligible studies. Second, some included RCTs may have small sample size. Third, some included studies may have poor methodological quality. Finally, there may be remarkable heterogeneity across the included trials. All those limitations may affect the findings of this study.

## Author contributions

**Conceptualization:** Ming-ming Fu, Xiang-dong Meng.

**Data curation:** Ming-ming Fu, Xiang-dong Meng.

**Formal analysis:** Ming-ming Fu, Xiang-dong Meng.

**Funding acquisition:** Ming-ming Fu.

**Investigation:** Xiang-dong Meng.

**Methodology:** Ming-ming Fu, Xiang-dong Meng.

**Project administration:** Xiang-dong Meng.

**Resources:** Ming-ming Fu, Xiang-dong Meng.

**Software:** Ming-ming Fu.

**Supervision:** Xiang-dong Meng.

**Validation:** Ming-ming Fu, Xiang-dong Meng.

**Visualization:** Ming-ming Fu, Xiang-dong Meng.

**Writing – original draft:** Ming-ming Fu, Xiang-dong Meng.

**Writing – review & editing:** Ming-ming Fu, Xiang-dong Meng.
